# Adapting a coach-assisted web-based intervention for parents of adolescents who refuse school: qualitative study with users of the Partners in Parenting Plus programme

**DOI:** 10.1192/bjo.2024.15

**Published:** 2024-03-15

**Authors:** Anna Smout, Glenn Melvin, Anthony Jorm, Marie B. H. Yap

**Affiliations:** Turner Institute for Brain and Mental Health, School of Psychological Sciences, Monash University, Melbourne, Victoria, Australia; School of Psychology, Deakin University, Melbourne, Victoria, Australia; Melbourne School of Population and Global Health, University of Melbourne, Victoria, Australia; Turner Institute for Brain and Mental Health, School of Psychological Sciences, Monash University, Melbourne, Victoria, Australia; and Melbourne School of Population and Global Health, University of Melbourne, Victoria, Australia

**Keywords:** School refusal, parenting, digital health, adolescent, internalising disorders

## Abstract

**Background:**

School refusal is a heterogenous problem which typically emerges in adolescence and co-occurs with internalising disorders. A substantial proportion of adolescents do not respond to existing treatment modalities; thus, novel, effective intervention options are needed. Partners in Parenting Plus (PiP+) is a coach-assisted, web-based intervention designed to empower parents to respond to adolescent internalising disorders.

**Aims:**

To conduct a process evaluation of PiP+ and identify programme adaptations required to meet the needs of parents of adolescents who refuse school.

**Method:**

Semi-structured interviews were conducted with 14 Australian mothers who had: (a) received the PiP+ programme (not tailored for school refusal) during a prior research trial; and (b) reported that their adolescent was refusing school during their participation in PiP+. Inductive thematic analysis was used to analyse interview transcripts.

**Results:**

Participants were 41–53 years old (*M* = 47.8) and parenting adolescent children aged 14–17 years (*M* = 14.9). Three themes illustrated how PiP+ features met or could better meet the needs of parents of adolescents who were refusing school: (a) feeling heard, supported and respected; (b) relevance to me and my context; and (c) seeing positive changes. Participants had favourable views of PiP+, especially coached components. Participants requested programme enhancements to better meet the needs of parents of neurodiverse adolescents and discussed the impact of cumulative help-seeking ‘failures’ on self-efficacy and locus of control.

**Conclusions:**

PiP+ was highly acceptable to the majority of parents navigating the issue of school refusal. This has implications for the enhancement of coach-assisted parenting interventions and the context-specific adaptation of PiP+ for school refusal.

School refusal, defined as persistent difficulty in attending school precipitated by significant emotional distress associated with attendance,^1^ is a heterogenous problem which requires a systems-level response. School refusal presentations typically emerge in early adolescence^2^ and usually involve comorbid anxiety and depressive disorders.^2,3^

Cognitive–behavioural therapy (CBT) is the most extensively evaluated treatment option for school refusal to date.^4^ However, one- to two-thirds of adolescents do not respond to this treatment modality.^5^ Parent-focussed interventions for school refusal have the potential to be as effective as targeting the adolescent directly^6^ and are therefore a viable alternative to adolescent-focussed treatments. Modifiable parent and family factors such as parent psychopathology, parental self-efficacy, maternal overprotection, family functioning and parent–child conflict have also been associated with school refusal.^7–11^ Although existing treatment programmes for school refusal include parent components,^4,5^ parents have rarely been centred as the primary focus of school-refusal intervention efforts. Moreover, parents face significant barriers to engagement in treatment offerings such as cognitive–behavioural therapy, which are usually conducted in a face-to-face format^12–14^ and conditional on the adolescent agreeing to participate in treatment. There exists a clear need for an innovative, parent-centred intervention option for school refusal which overcomes barriers to parental engagement.^15^ One strategy to enhance intervention engagement is the use of digital technologies in intervention delivery.^16^ Evidence from meta-analyses and randomised trials supports the efficacy of parenting interventions delivered online in achieving the intended parent- and child-level outcomes,^17^ to a similar or equivalent extent to in-person delivery.^18,19^

Partners in Parenting Plus (PiP+), formerly known as the Therapist-Assisted Online Parenting Strategies program (TOPS),^20^ is a multi-level, manualised, web-based parenting approach to prevention and early intervention for adolescent internalising problems.^21^ A full description of the multi-level PiP programme is provided in ref. ^21^. Across the four levels of PiP, parents are provided with increasing levels of support to align their parenting behaviours with a set of expert-consensus and evidence-based parenting guidelines on how to prevent and respond to adolescent anxiety and depression.^22^ PiP+ constitutes level 4 of PiP and is designed to meet the needs of parents of adolescents with clinically significant internalising problems by supplementing online material with one-on-one coaching sessions delivered via video conference.^20,21^ PiP+ is ideally placed for further adaptation in the context of school refusal owing to considerable overlap between parent and/or family factors associated with school refusal and internalising disorders,^7,9,10,23^ which are addressed in the existing PiP+ intervention. However, little is currently known about the specific needs of parents of adolescents who refuse school in the context of internalising disorders and how these could be met by a parenting programme such as PiP+.

## The present study

The present study contributes to the process evaluation and context-specific development of PiP+ for school refusal, through forward and backward evaluation.^24^ In forward evaluation, stakeholder feedback regarding a future goal is gathered. Backward evaluation considers the extent to which intended programme outcomes were achieved.^24^ Stakeholder evaluation expedites closeness of fit between the programme and stakeholder needs, and programmes which are highly acceptable to stakeholders are more likely to reach their potential in terms of engagement and effectiveness.^24–26^ The primary aim of this study was therefore to identify programme adaptations required to meet parent needs according to the lived experience of parents who (a) had received the original PiP+ programme that was not tailored for school refusal; and (b) reported that their adolescent was struggling with school attendance during their participation in PiP+. A secondary aim of the present study was to ascertain the perceived effectiveness of PiP+ in achieving its intended outcomes in this sample.

Qualitative methods were used to answer the following research questions. (1a) How, if at all, did the PiP+ programme meet parents’ needs in responding to school-refusal problems in their adolescent? (1b) How, if at all, did the PiP+ programme meet parents’ needs in responding to clinical-level anxiety or depression in their adolescent? (1c) How could the PiP+ programme be adapted to better meet the needs of parents responding to adolescent school refusal, anxiety and depression? In general, (2a) what changes in parenting practices, if any, were made as a result of participating in the PiP+ programme; and (2b) what perceived impact, if any, did any changes in parenting practices have on the adolescent?

## Method

### Study design and approach

We chose qualitative methods for this study owing to their suitability for process evaluation research^27^ and their value in adapting interventions to suit a specific context.^28^ A phenomenological approach was adopted to develop a rich understanding of how the PiP+ programme was experienced and could be enhanced.^29^ Braun & Clarke's inductive approach to reflexive thematic analysis^30,31^ was used to analyse interview transcripts within a critical-realist framework. Based on principles of data saturation and the specificity of the parent experience under study, we estimated that a sample size of 12–14 participants (to be confirmed once data collection was underway) would be adequate to generate a sufficiently rich account of participant perspectives regarding PiP+.^32–34^

### Participants

All parent participants of the original PiP+ trial who, during the trial, had reported that their adolescent had been struggling with school attendance in the context of their internalising difficulties (*N* = 23) were eligible to participate. A stratified purposive sampling strategy was used to incorporate heterogeneity in characteristics relevant to parents’ experience of the programme.^35^ These characteristics were (a) severity of school attendance difficulties experienced during the original PiP+ trial (coded as mild/moderate versus moderate/severe; ascertained qualitatively by reviewing recorded PiP+ coaching sessions in consultation with G.M., a senior clinical psychologist with clinical and research expertise in school refusal); (b) parents’ improvement in their concordance of parenting practices with a set of evidence-based parenting guidelines (primary outcome of the original trial;^36^ coded as showing improvement or no improvement in concordance scores); and (c) adolescent-report anxiety scores as measured at baseline using the Spence Children's Anxiety Scale^37^ (scores coded as falling above or below the clinical threshold).^38^ The cut-off points were as follows: Spence Children's Anxiety Scale, boys ≥33, girls ≥40;^34^ Short Mood and Feelings Questionnaire, ≥12.^39^

In total, 21 parents were invited to participate. Six did not respond, and one replied affirmatively via email but did not respond to further contact. No participants were excluded following screening. The final recruited sample (*N* = 14) all identified as women, were aged between 41–53 years and lived in Australia. Sample characteristics are provided in Table 1.

**Table 1:** Sample characteristics of interview participants and their adolescents (*N* = 14)

Variable	Descriptor	
Parent characteristics (pre-intervention)		
Gender; *n* (%)	Woman	14 (100%)
Parent age in years, mean (range)		47.8 (41–53)
State/territory, *n* (%)	Victoria	11 (79%)
	Australian Capital Territory	2 (14%)
	Queensland	1 (7%)
Relationship to adolescent, *n* (%)	Mother	14 (100%)
Relationship status, *n* (%)	Single	1 (7%)
	Married/de facto	10 (71%)
	Separated or divorced	3 (21%)
Caring arrangements, *n* (%)	Child living with both parents/guardians	8 (57%)
	Parents separated but both involved in adolescent's care	2 (14%)
	Parents separated with only participating parent involved in adolescent's care	4 (29%)
Highest level of education, *n* (%)	Trade/apprenticeship	1 (7%)
	Technical certificate	1 (7%)
	Diploma	3 (21%)
	Bachelor degree	3 (21%)
	Postgraduate degree	6 (43%)
Adolescent characteristics (pre-intervention)		
Gender, *n* (%)	Girl/woman	10 (71%)
	Boy/man	4 (29%)
Adolescent age in years, mean (range)		14.9 (14–17)
Adolescent living arrangements, *n* (%)	Living with both parents/guardians	9 (64%)
	Living with participating parent/guardian only	5 (36%)
Schooling format, *n* (%)	In-person attendance	11 (79%)
	Virtual school or distance education	3 (21%)
Adolescent self-report symptom elevation: anxiety (SCAS), *n* (%)	Above clinical cut-off	8 (57%)
	Below clinical cut-off	3 (21%)
	Missing	3 (21%)
Adolescent self-report symptom elevation: depression (SMFQ), *n* (%)	Above clinical cut-off	7 (50%)
	Below clinical cut-off	4 (29%)
	Missing	3 (21%)
Sampling characteristics		
PRADAS improvement (score at baseline versus post-intervention), *n* (%)	Yes	7 (50%)
	No	7 (50%)
Qualitative school-refusal severity classification, *n* (%)	Mild/moderate	6 (43%)
	Moderate/severe	8 (57%)
Adolescent self-report SCAS score (pre-intervention)	Mean (range)	52.5 (17–85)
	Missing (*n*)	3
Parent-report CALIS-SR score, mean (range)^a^		40.2 (17–53)

SCAS, Spence Children's Anxiety Scale; SMFQ, Short Mood and Feelings Questionnaire; PRADAS, Parenting to Reduce Adolescent Depression and Anxiety Scale; CALIS-SR, Child Anxiety Life Interference Scale, adapted for school refusal.

a. Gathered during screening call.

### Procedure

**Fig. 1:**
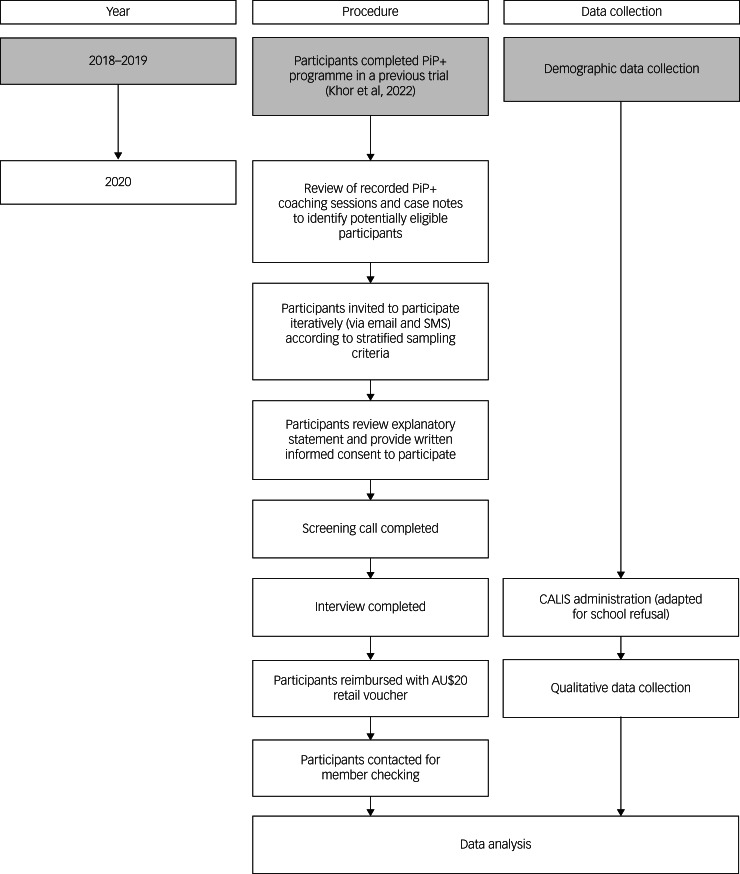
Flow chart of study procedure and data collection processes. Grey cells indicate research activities conducted in the first Partners in Parenting Plus (PiP+) trial.^36^ CALIS, Child Anxiety Life Interference Scale; SMS, short message service.

A flow chart illustrating the study procedure and data collection processes is provided in Fig. 1. Recordings of coaching sessions and corresponding case notes during the PiP+ trial were reviewed by A.S. to identify eligible participants. Parents who were identified as eligible were invited to participate via email and short message service (SMS), approximately 1 year following completion of the PiP+ intervention. After reviewing the explanatory statement and providing written consent, parents were contacted to schedule a screening call and interview over Zoom video conferencing software. Participants were reimbursed for their time with a AU$20 retail voucher. After transcription processes were complete, all participants were contacted for member checking. Six participants were provided with copies of their transcripts and/or audio files to review, though none responded with corrections, clarifications or additions. All procedures complied with the Helsinki Declaration of 1975 (as revised in 2008) and were approved by the Monash University Human Research Ethics Committee (ID: 11095).

### Data collection

Demographic data had been previously obtained from the participating parents during the registration phase of the PiP+ trial. For the present study, data were collected during one-on-one audio- and video-recorded screening calls and semi-structured interviews, all conducted in English by A.S. (MPhil, PhD (Clinical Psychology) candidate and provisional Psychologist, female). A.S. did not have established relationships with any participants prior to study commencement.

Screening calls were conducted to anchor the parent back to the timeframe within which they engaged with PiP+ and discuss the presence and/or degree of school refusal experienced by the adolescents during their parents’ engagement with PiP+. An adapted version of the parent-report Child Anxiety Life Interference Scale^40^ was also administered during the screening call, with the questions displayed on screen. This validated measure of life interference associated with child/adolescent anxiety was adapted by the authors to assess life interference related to school refusal by replacing all references to anxiety with school refusal.

To assess parent perspectives, a PiP+ evaluation interview (semi-structured interview schedule, available on request) was developed and piloted by the authorship team. Parents were asked to share their experiences of how the PiP+ programme had or had not met their needs related to parenting an adolescent who was refusing school, and how the programme could be enhanced to this end. The interview also sought to understand what changes, if any, were made to parenting practices as a result of programme participation. Participants were encouraged to talk freely about the challenges of parenting an adolescent who was refusing school, with prompts (e.g. ‘Can you say more?’, ‘Why is that important to you?’) used to deepen responses and facilitate exploration of ideal programme attributes. Data collection stopped when adequate data had been collected to address the research questions in depth.^32^ No repeat interviews were required.

### Data analysis

Descriptive statistics of demographic data and responses to the adapted version of the Child Anxiety Life Interference Scale^35^ were conducted using IBM SPSS version 28.0.^41^ All interviews were transcribed verbatim and de-identified by A.S. (first author) or by a trained research assistant before being reviewed for accuracy by the first author. Coding and thematic analysis were conducted by the first author adhering, to Braun & Clarke's six-phase, reflexive application,^30,42^ using NVivo 11.^43^ The six phases consisted of (a) data familiarisation and immersion through transcription, noting down initial coding ideas during repeated reading of transcripts and corroboration with field notes; (b) inclusive inductive generation of preliminary codes; (c) generating initial themes by sorting codes into candidate themes and subthemes; (d) review and refinement of initial themes; (e) naming and defining themes; and (f) final analysis and write-up of the data narrative.^42^ Themes were recursively constructed and reviewed and were conceptualised as ‘stories about particular patterns of shared meaning across the dataset’^30^ (p. 952). All authors contributed to the review and refinement of initial themes. Material was only excluded if it was irrelevant to the research questions or inaudible.

## Results

**Fig. 2:**
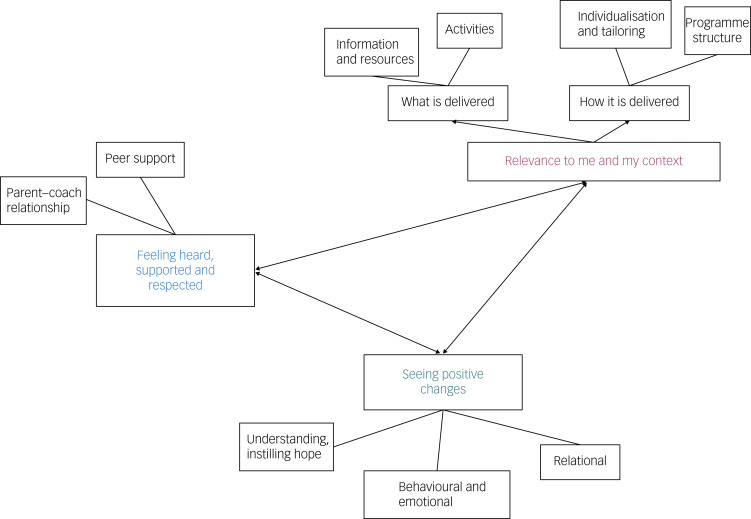
Thematic map illustrating theme and sub-theme classifications and the relationships between themes.

Participants were 41–53 years old (*M* = 47.8) and parenting adolescent children aged 14–17 years (*M* = 14.9). Screening calls lasted between 25 and 55 min (mean: 36.8 min). Interviews were conducted between September and December of 2020, approximately 2 weeks after the screening call, and lasted between 38 and 68 min (mean: 49.8 min). Three themes were identified from interview data which illustrated the ways PiP+ features met or could better meet the needs of parents of adolescents who were refusing school, and how the programme could be enhanced to this end: (a) feeling heard, supported and respected; (b) relevance to me and my context; and (c) seeing positive changes. These themes represent central organising concepts for 11 related subthemes. Definitions of each theme are provided in Table 2, and a visual representation of the themes and subthemes is provided in Fig. 2. Under each theme, general experiences and challenges shared by parents which add richness and context to their perspectives are discussed, with corresponding programme-specific comments explained in each subtheme. Throughout all quotes, names have been replaced with pseudonyms marked with an asterisk, and references to ‘TOPS’ by participants relate to PiP+.

**Table 2:** Definitions of overarching themes

Theme	Definition
I feel heard, supported and respected	An overarching theme that explains the ways PiP+ did or did not meet the connection and emotional support needs of parents, what these needs are, and how the programme could be enhanced to this end. It includes the ways the parent–coach relationship and coach qualities affected parents’ experience of the programme and how peer support options could further increase the benefits experienced by parents.
It's relevant to me and my context	An overarching theme that explains how the programme content (what is delivered) and design (how it is delivered) both affected and could be adapted to augment the perceived relevance of the programme to parents’ individual preferences and context. It includes the way individualisation, tailoring and programme structure contributed to parents’ experience of the programme and could be adapted to suit the needs of parents of school-refusing adolescents. It also includes parent suggestions about content (strategies and skills, information and resources, activities) to retain, add, change or remove.
I see positive change	An overarching theme which explains the direct and indirect effects of programme participation that were perceived and/or desired by parents. It includes the importance of understanding and instilling hope (related to the issue of school refusal and how it can be overcome), as well as the behavioural, emotional and relational changes in themselves, their teen and their parent–child relationship that parents believed were positive and/or important for the programme to facilitate. This theme additionally includes barriers and enablers parents experienced with respect to adjusting their parenting practices according to programme recommendations.

### Feeling heard, supported and respected

Participants described common emotional experiences of isolation, shame, helplessness and distress when reflecting upon the challenges of responding to their adolescent's school refusal:

‘I'd driven to school with Vanessa* in the car and she was in a fetal position crying. “I can't get out of the car, I don't want to go, I don't want to go, don't make me go”, and I'm in the front seat going … I can't even get my daughter out of the car … I just went right, I've had enough, we can't do this … the distress, for me, for her, it's too much, it's inhuman, I can't keep dragging my child up here with all this stress, and there's no way forward … when your child won't get out of the car … ’ [Participant 10].

In light of such experiences, participants discussed the value of the parent–coach relationship they developed in PiP+ and suggested that peer support options may provide additional benefits.

#### Parent–coach relationship

All participants commented that the coaching sessions were the most beneficial aspect of the programme, owing to the qualities of their coach and the therapeutic processes arising from one-on-one engagement: ‘The support for me was invaluable, because with that support I got more confidence … it was a release for me too, and then I was better able to be de-stressed. That's important, parents need to talk to somebody, and not feel like failures’ [Participant 11]. Participants also valued the personalisation of the programme content to their own individual context: ‘Because it was a one-on-one session … we were able to individualise the programme for our situation … just using the basic framework of the TOPS [PiP+] programme’ [Participant 4].

#### Peer support

A subset of participants suggested peer support avenues would be a helpful addition to PiP+, to further combat feelings of isolation related to school refusal: ‘I didn't know any other parents who have these issues so I felt really alone … perhaps a community for parents to support each other would be helpful too’ [Participant 12]. Overall, however, there was low consensus and specificity as to what form peer support should take if included. Among the suggestions were moderated online group check-ins and connecting parents to existing peer support networks outside of PiP+. Others felt that participating in PiP+ had adequately conveyed that they weren't alone in facing the issue.

### Relevance to me and my context

Participants frequently referred to the heterogeneity in the needs of different families and the value of this being catered to in programme content and delivery. Regarding challenges relevant to school refusal, most had experienced this issue being misunderstood among school and parent populations, with consequent barriers to obtaining support: ‘I didn't really understand about anxiety and mental health wasn't something I'd had a lot to do with … even teachers you know, “oh we're just not tough enough” … no, actually this is a really serious thing, and we need to work through it in a compassionate way’ [Participant 10]. They also noted significant gaps in their own knowledge about alternative schooling pathways available, where to find expert help and how to approach school staff. They described lacking knowledge about what could reasonably be asked of school staff, reluctance to add to a teacher's load and indirect parent–teacher communication methods as barriers to connecting with school staff in support of their adolescent.

Participants further discussed neurodevelopmental difficulties as an additional obstacle to receiving tailored support, within PiP+ and beyond. In light of such experiences, participants provided suggestions for improving programme content and delivery mechanisms to meet their individual needs. These are elaborated in the following sections.

#### How it is delivered

##### Individualisation and tailoring

Most parents were satisfied with the broad content coverage of PiP+ and the degree of tailoring of content to their own context facilitated by their coach: ‘Because it was a one-on-one session with a trained psychologist we were able to, individualise the programme for our situation’ [Participant 4]. Parents hoped that future school refusal-specific content would be similarly individualised to the needs of each family. A subset of participants commented that tailoring could have been further augmented, for example, by incorporating opportunities to revisit content: ‘I think each family person [sic] could be offered an opportunity to revisit one or two topics, and they can nominate what they are’ [Participant 3]. Parents of neurodiverse adolescents commented on a relative lack of tailoring to such circumstances in PiP+. To overcome this, screening or assessment for neurodevelopmental disorders was proposed as a helpful inclusion: ‘I think doing a survey … are these issues, does your child have difficulties or is … not open to wearing certain things or do they refuse to … certain things that are common things that happen with kids that come under this kind of umbrella’ [Participant 9].

##### Programme structure

The structure of the PiP+ programme was described as acceptable and beneficial to parents for several reasons, including (a) the combination of self-led and coach-assisted components enhancing learning by reinforcing key messages and placing concepts into the parent's context; (b) the positive sense of accountability instilled in parents by coach-supported goal setting activities; and (c) how, by opting into the time-limited programme, parents prioritised reflecting on their parenting. Conversely, one parent highlighted the challenges to weekly engagement and progress posed by shared custody arrangements and difficulty achieving early milestones: ‘I always felt a bit like a failure when I didn't, you know, achieve my goals cause I couldn't get Jay* to talk to me … I only have him half the week and so yeah, sometimes I'd get to the next appointment and it's like ‘oh we haven't managed to talk about this’ [Participant 8].

Regarding school refusal, there was strong consensus among participants that adolescent well-being and parent–adolescent relationships should be prioritised before addressing school attendance. For this reason, they endorsed the original PiP+ topic structure and advocated for it to be retained in the new programme. There was broad agreement that the issue of school refusal should be covered in a dedicated module, as well as being incorporated thematically throughout the programme.

#### What is delivered

##### Information and resources

Parents advocated for others responding to school refusal being made aware of the mental health underpinnings of the issue, alternative learning pathways available and how to form constructive school–family relationships. The majority said the existing PiP+ content directly or indirectly assisted them in responding to their adolescent's school refusal. However, one participant experienced the content as being too generic for their needs. Most felt they had learnt something new from participating in PiP+; even if they held pre-existing knowledge about content included in the programme, having this affirmed by their coach, and by current research, contributed to an increase in their parenting confidence. ‘It helped me to realise that I was on the right track … that there was research supporting it, it just helped give me confidence and backed up what I was doing’ [Participant 1]. ‘It was quite basic level stuff … but by discussing it again and reviewing it and applying it I definitely learned a lot’ [Participant 6].

To support consolidation of learning, parents also suggested the provision of printable topic tip-sheets to review key information with ease.

##### Activities

Participants relayed how opportunities for guided reflection facilitated by their PiP+ coach had improved their understanding of the factors contributing to their teens’ difficulties, including the impact of their own parenting behaviour. Most agreed that future parents would therefore benefit from similar guidance to explore the underlying causes of their adolescent's school refusal: ‘What's causing the school refusal you know is it, a social issue is it, struggling with the workload is it … bullying is it, previous experience … and also dig a bit too in learning disabilities’ [Participant 5]. Participants also cared about the inclusion of activities that supported their learning and engagement. They favoured case studies, hypothetical scenarios, voice recordings and quizzes.

### Seeing positive changes

Participants discussed emotional and relational challenges associated with parenting an adolescent who is struggling with their mental health and/or refusing school, such as feelings of hopelessness, panic, self-blame and disconnection. In this context, they discussed their experiences of how (if at all) the PiP+ programme enhanced their understanding of their adolescent's perspective, hope for the future, and any positive behavioural, emotional or relational changes they observed in themselves, their adolescent and their relationship, as a result of their participation in PiP+. Parents acknowledged that such changes had required their time, effort and persistence: ‘I wanted instant gratification and acknowledgement, but when I learned to cool my heels it took six months to eight months after, for the feedback [some positive reinforcement] to come’ [Participant 3].

Participants also discussed the barriers and enablers they experienced with regard to making changes to their parenting in accordance with the recommendations of the PiP+ programme.

#### Understanding, instilling hope

As previously mentioned, experiencing increased understanding of the factors underpinning their adolescent's symptoms and behaviour through participation in PiP+, and the impact of this on their parenting, resonated across several individuals. Parent commentaries conveyed how reinterpreting school refusal as a manifestation of anxiety, rather than parenting failures or adolescent defiance, had led to increased self-compassion and hope for the future, and a reduction in self-blame. ‘Sometimes things can happen in someone's life that derails them, but it doesn't mean that it's permanent … whatever has happened is valid for that person, and there are ways around’ [Participant 12].

These parents were more likely to express sentiments indicative of increased parental self-efficacy (belief that their behaviour could effect positive change for their adolescent) and motivation to persevere. The inverse was true among some parents who continued to face barriers in identifying the underlying causes of their adolescent's mental health and school attendance difficulties. This was more likely to occur if difficulties were chronic, perceived as less responsive to parental efforts and/or further complicated by neurodevelopmental comorbidities. Parents in these circumstances were more likely to convey feelings of self-blame, regret and hopelessness: ‘I almost feel like I need to … go on a training course to understand how to decode the kids, and I, I feel like I've really let Natalia* down’ [Participant 12].

#### Behavioural and emotional

The majority of parents reported making adjustments to their parenting behaviours as a result of participation in PiP+, including actively listening, validating and empathising with their adolescent more frequently, prioritising self-care, and promoting their adolescents’ autonomy and supporting them to face fears: ‘Instead of just going “okay let's not do that that's terrifying thing” going “well, yes I know it's terrifying but we can do it together”’ [Participant 5]. Most parents also described feeling better equipped to remain calm during stressful parenting situations, owing to increases in their parenting self-awareness and confidence: ‘I was less reactive … by managing my emotions better I was better able to talk to Andreas* more calmly, so then he could probably be more receptive to my advice’ [Participant 11]. Over time, some parents believed the changes they made to their parenting contributed, at least in part, to their adolescents becoming more calm and confident and less angry and afraid. Some also observed their adolescent to be more communicative and social with family and peers, and less withdrawn.

A subset of parents explained that the perceived benefit of the programme for them was attenuated because of chronic and/or severe school refusal and mental disorder histories, with long-standing and ongoing ambiguity regarding the causes of the problem. These parents often situated such reflections within the timing of their engagement in PiP+, relative to the onset of their adolescent's difficulties and/or their achieving diagnostic clarity about their adolescent's symptoms. Parents who described a mismatch between the timing of their PiP+ engagement and diagnostic clarity were more likely to describe struggling to implement the strategies endorsed throughout the programme: ‘The reason the programme wasn't helping is because Jay*'s autistic and I didn't know it … so, yeah back then when we were talking about connection … I was cognisant that I could not connect with Jay*, but autism never entered my mind’ [Participant 8].

Parents in this subset also explained they had not made changes to their parenting owing to sustained doubt that this could significantly contribute to improvements in their adolescent. Such beliefs were described to occur against a background of ongoing barriers to obtaining effective individual treatment for their adolescent in the past: ‘She's been seeing paediatricians since three and a half … If I'd have been armed with this information at a younger age I could have … got her the help that she needed, and it could have completely changed her whole schooling’ [Participant 9]. In this context, parents were more likely to express sentiments reflective of a developed external locus of control when it came to the impact of their parenting on their adolescent: ‘We had tried a lot of the stuff too with the psychologist as well, so … at the end of the day it was up to her [adolescent])’ [Participant 13].

In response, participants advocated for future programme iterations being accessible to parents as soon as the issue emerges and, in the case of school refusal, immediately available at the recommendation of school staff.

#### Relational

Several parents observed that making adjustments to their parenting contributed to significant improvements in connectedness with their adolescent. Some perceived their adolescent to be more trusting of them and more likely to approach and confide in them, owing to feeling more respected and supported overall: ‘He sort of seeks us out every now and again to spend a bit of time with him … I think he trusts me a lot more than he did before, cause he can see that I'm taking into account his perspective’ [Participant 4].

## Discussion

This study contributes to the process evaluation and context-specific development of PiP+ for parents of adolescents who refuse school in the context of internalising disorders. The study also provides insight into the experiences and needs of such parents, as related to the enhancement of an online parenting programme. Regarding the challenges of parenting an adolescent struggling with school refusal and their mental health, 14 PiP+ programme participants conveyed the value of (a) feeling heard, supported and respected throughout programme engagement; (b) the programme content and delivery processes being relevant and tailorable to their needs and context; and (c) the perceived direct (parent) and indirect (adolescent) effects of PiP+.

Overall, the existing PiP+ programme content and structure were strongly aligned with the needs of parents of adolescents who refused school. Parents were unanimous that the coaching sessions were the most beneficial aspect of the PiP+ programme. The reasons for this included being heard and understood while discussing stressful parenting experiences and the tailoring of programme content to their own context facilitated by their coach. Many parents highlighted the relevance of existing PiP+ content to the issue of school refusal, particularly parenting strategies to strengthen parent–adolescent connection and communication, respond to anxiety and mitigate conflict. Parents also advocated a retention of the current PiP+ topic structure, which prioritises parent–adolescent relationships and psychoeducation ahead of tackling more specific issues such as school refusal. The provision of a parenting coach appropriately matched the support and engagement needs of those parents who explained that the online content alone would not have been sufficient. The finding that peer support did not constitute a predominant theme in this study may have been due to the degree and quality of socioemotional support parents received from their PiP+ coach.^44^

Nonetheless, parents identified ways that PiP+ could be enhanced to meet additional needs related to information provision and tailoring of content. First, participants believed that increasing parent understanding of the possible causes of and maintenance factors in their adolescent's school refusal should be prioritised in any future iteration of PiP+ targeting school refusal. They also highlighted the importance of receiving practical guidance to effectively partner with school staff in support of their adolescent's attendance goals. Further investigation into mutually acceptable approaches to the establishment of effective school–family partnerships is therefore an important future direction for this line of enquiry. Second, the extent to which PiP+ was described to have met parent needs among those parenting an adolescent with neurodevelopmental comorbidities varied between participants. Whereas some parents experienced the programme as being beneficial to them despite the lack of tailoring to the circumstances of neurodiverse adolescents, others experienced the programme content as less directly applicable and implementable in this context. Indeed, the need for programme adaptations among specific vulnerable subgroups, including parents of neurodiverse adolescents, was anticipated in the development of the multi-level PiP model.^21^ The perspectives shared in the current study confirm that PiP+ is an acceptable platform to build upon in this manner. In addition, enquiring about prior diagnoses (including neurodevelopmental disorders) during registration could facilitate the PiP+ coach to most optimally tailor the PiP+ content to neurodiverse presentations from the outset. Content addressing challenges to implementing recommended parenting strategies where an adolescent has a neurodevelopmental disorder would also be likely to be valuable, as would information about accessing neurodevelopmental assessments where indicated. The feasibility of including formal screening for neurodevelopmental disorders, as requested by parents, would depend on the implementation setting.

Finally, important qualitative insights were gleaned about what changes to parenting practices were made by parents, the perceived impact of such changes on the adolescent and the barriers to and/or facilitators of these. The majority of participants reported making changes to their parenting as a result of their PiP+ participation. Consistent with results of the quantitative programme evaluation,^36^ parents described making changes concordant with parenting guidelines to prevent and respond to adolescent anxiety and depression,^22,45^ sustaining changes long-term, with their adolescent being responsive to their efforts (albeit after 6–12 months of parent persistence with strategy implementation) in the form of observed emotional, behavioural and relational improvements. However, a subset of parents reported that they did not make changes to their parenting as a result of PiP+ participation. Longstanding mental health and school refusal difficulties, diagnostic ambiguity and unmet treatment needs seemed to render some parents vulnerable to deeper-rooted beliefs of futility regarding their own and/or others’ attempts to effect positive change. It follows that parents experiencing disempowerment to this extent may benefit from support to directly identify and address any unhelpful cognitions and appraisals related to help-seeking and parenting, which may otherwise continue to attenuate the degree to which they engage with and benefit from the support offered.^39,46–49^ In sum, the programme content was perceived by parents as most useful and appropriate for early intervention with clinically significant internalising problems, consistent with the premise of the PiP multi-level programme design.^21^

### Limitations

The findings of the present study must be interpreted in light of some limitations. Parent perspectives were gathered approximately 1 year after participation in PiP+; thus, the perspectives shared are vulnerable to recall bias. Second, the present sample all identified as female, were highly educated, and were drawn from a motivated, help-seeking sample; thus, generalisability is limited. However, good variance across factors that were anticipated to be relevant to parent experience of the programme was achieved using stratified purposive sampling methods (Table 1) and, despite the specific sample characteristics required for this study, a good sample size was obtained (*N* = 14). In addition, an inclusion criterion for participation in the PiP+ trial,^36^ from which the current sample was drawn, was for the adolescent of the participating parent to be actively engaged with a mental health professional. As such, any parent-reported impacts of the PiP+ programme on adolescent functioning may have been affected by any individual treatment the adolescent had received. It is conceivable that the degree of improvement (if any) that parents perceived in their adolescent since their PiP+ participation proportionately coloured their retrospective appraisals of the programme.

In conclusion, this qualitative study gathered consumer-driven, context-specific directives for adaptations and enhancements to PiP+ by drawing upon the lived experience of a sample of parents who had received the programme and, during their participation, reported that their adolescent had refused school in the context of internalising difficulties. This study also provides in-depth insight into the perceived long-term effectiveness of the PiP+ programme in producing intended outcomes, and potential barriers to this. In addition, the parenting-related experiences, challenges and needs of those parenting an adolescent who is refusing school were shared. A key finding of the present study was that the current design of the PiP+ programme, particularly the provision of one-on-one coaching support, was highly acceptable to the majority of the interviewed parents. This outcome reinforces the findings of the first evaluation of PiP+, which saw high rates of intervention engagement, low attrition and large intervention effects on parent outcomes.^36^ Important additions to the programme were identified, which included the presentation of factors known to contribute to the development and maintenance of school refusal and the provision of guidance for parents to work together with their adolescent's school staff in support of their attendance.

The present findings draw attention to the need to consider programme enhancements which cater to the needs of parents of adolescents living with neurodiversity, namely, an augmented screening process to facilitate the optimal tailoring of content to parent circumstances by the coach from the outset. Moreover, this study highlights the way cumulative help-seeking ‘failures’ prior to programme engagement may meaningfully hinder the extent to which parents benefit from coach-assisted parenting interventions, unless addressed. The majority of parents perceived the programme to have produced intended outcomes, and their reflections support the need for long-term follow-up of adolescent outcomes of parenting interventions, which they observed to take hold 6–12 months post-intervention. Overall, these findings could inform the enhancement of coach-assisted parenting programmes and the development of PiP+ for school refusal, as well as ongoing hypothesis testing regarding facilitators of and barriers to the achievement of intended outcomes among parenting interventions targeting adolescent internalising disorders.

## Data Availability

A summary of the data that support the findings of this study are available on request from the corresponding author, M.B.H.Y. The data are not publicly available owing to their containing information that could compromise the privacy of research participants.
